# Identification of the antibacterial mechanism of cryptotanshinone on methicillin-resistant *Staphylococcus aureus* using bioinformatics analysis

**DOI:** 10.1038/s41598-021-01121-9

**Published:** 2021-11-05

**Authors:** Jiwei Zhong, Haidan Wang, Yun Zhuang, Qun Shen

**Affiliations:** 1 Department of Emergency Medicine, Affiliated Hospital of Nanjing University of Chinese Medicine, Nanjing, 210029 China; 2 Department of Pharmacology, Affiliated Hospital of Nanjing University of Chinese Medicine, Nanjing, 210029 China; 3 Department of Hematology, Affiliated Hospital of Nanjing University of Chinese Medicine, Nanjing, 210029 China

**Keywords:** Computational biology and bioinformatics, Microbiology

## Abstract

Cryptotanshinone (CT) is an extract from the traditional Chinese medicine *Salvia miltiorrhiza*, which inhibits the growth of methicillin-resistant *Staphylococcus aureus* (MRSA) in vitro. This study aims to determine the antibacterial mechanisms of CT by integrating bioinformatics analysis and microbiology assay. The microarray data of GSE13203 was retrieved from the Gene Expression Omnibus (GEO) database to screen the differentially expressed genes (DEGs) of *S. aureus* strains that were treated with CT treatment. Gene ontology (GO) and the Kyoto Encyclopedia of Genes and Genomes (KEGG) pathway enrichment analyses were used to identify the potential target of CT. Data mining on the microarray dataset indicated that pyruvate kinase (PK) might be involved in the antimicrobial activities of CT. The minimum inhibition concentrations (MICs) of CT or vancomycin against the MRSA strain ATCC43300 and seven other clinical strains were determined using the broth dilution method. The effects of CT on the activity of PK were further measured. In vitro tests verified that CT inhibited the growth of an MRSA reference strain and seven other clinical strains. CT hampered the activity of the PK of ATCC43300 and five clinical MRSA strains. CT might hinder bacterial energy metabolism by inhibiting the activity of PK.

## Introduction

One of the largest concerns in public health is the continual emergence of multidrug-resistant bacterial pathogens, which severely limits treatment options. *Staphylococcus aureus* is a particularly problematic pathogen that is prevalent in human and animal populations. This organism commonly causes infections of the superficial skin, soft tissue, surgical wounds, and sometimes the bloodstream and lungs. Methicillin-resistant *S. aureus* (MRSA) and specific strains with reduced susceptibility to vancomycin could cause infections and diseases that are difficult to treat or resistant to the empirical antibiotics^1^. The global supply of antibiotics that are available for the treatment of infections that are associated with this microorganism is decreasing.

Many studies have reported that medicinal herbs from different countries exhibited anti-MRSA activities, which was due to their phytochemical contents^2^. These plants could be employed as alternatives for drug development to stop, or control, or both MRSA infections.

Cryptotanshinone (CT) is a fat-soluble extract from the traditional Chinese medicine *Salvia miltiorrhiza* (Danshen), which dilates blood vessels, and has antitumor and antiinflammatory activity^3^. In addition, CT inhibits the growth of *S. aureus* and MRSA in vitro^4^; however, the mechanism of action is unknown, which limits its further applications.

Microarray platforms were recently employed to understand gene expression in bacteria that were treated by various Chinese medicines to identify some pathogen genes that are associated with antibacterial mechanisms^5^. In addition, the Gene Expression Omnibus (GEO) database offers methods for the downstream bioinformatics mining of gene expression profiles in a variety of bacteria.

In this study, the differentially expressed genes (DEGs) were identified between strains of *S. aureus* that were treated with normal saline and CT by mining microarray datasets from the GEO database, which aimed to identify and confirm the mechanism associated with the antibacterial effects of CT.

## Results

### DEGs

**Figure 1:**
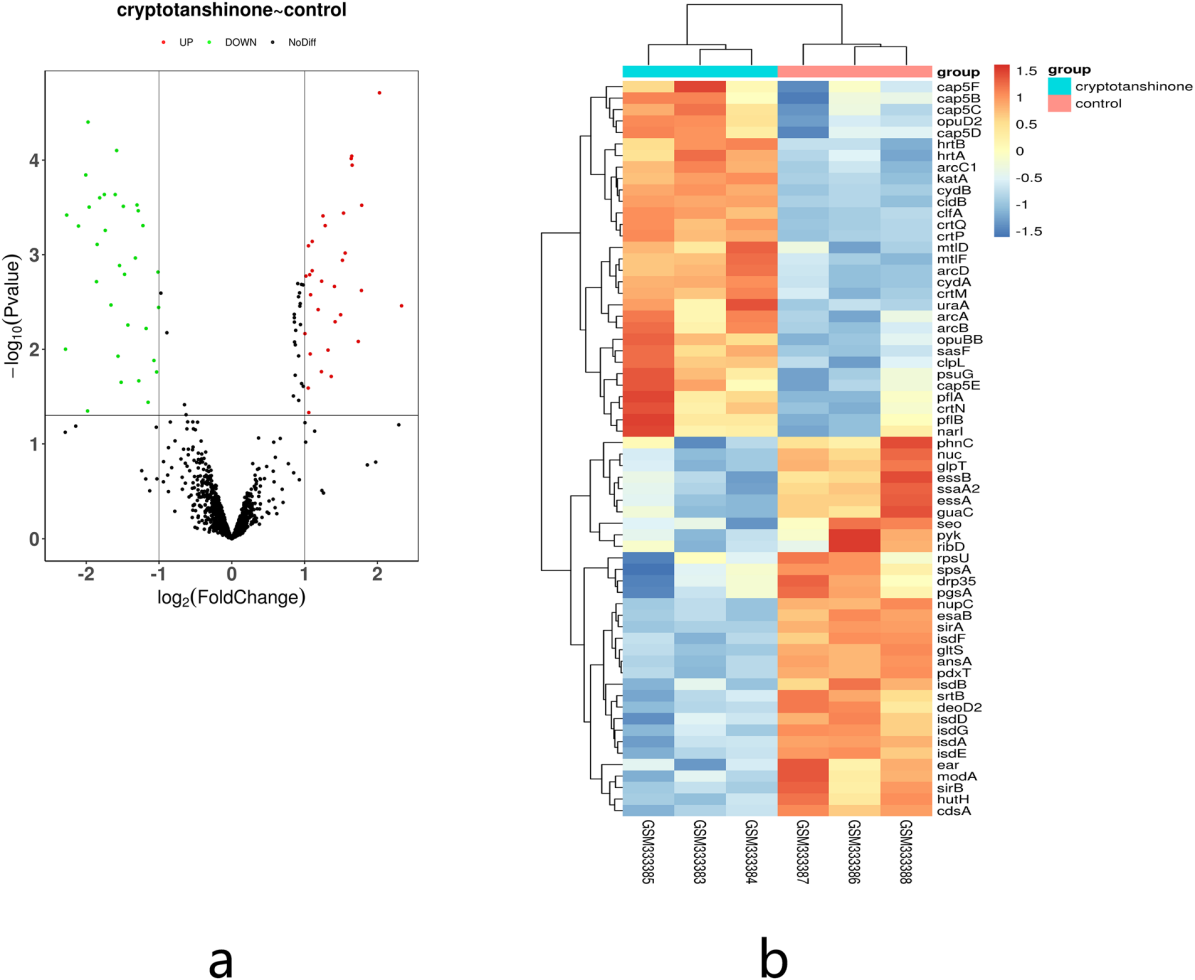
Screening of DEGs in MRSA induced by CT treatment: (**a**) volcano plots; and (**b**) hierarchical cluster analysis (heatmaps) of the common DEGs between CT treated samples and normal controls in GSE 13203. Statistically significant DEGs were defined with *p* < 0.05 and absolute log_2_FC > 1.0 as the cutoff threshold.

The volcano and heatmaps plots were generated to show the down and upregulated genes in the GEO datasets (GSE13203). A total of 64 overlapping DEGs (absolute log_2_FC > 1 and FDR < 0.05) were identified, which included 33 downregulated and 31 upregulated genes, respectively (Fig. 1). The top ten up and downregulated DEGs are listed in Table 2.

### GO and KEGG pathway of DEGs enrichment analysis (Fig. 2)

**Figure 2:**
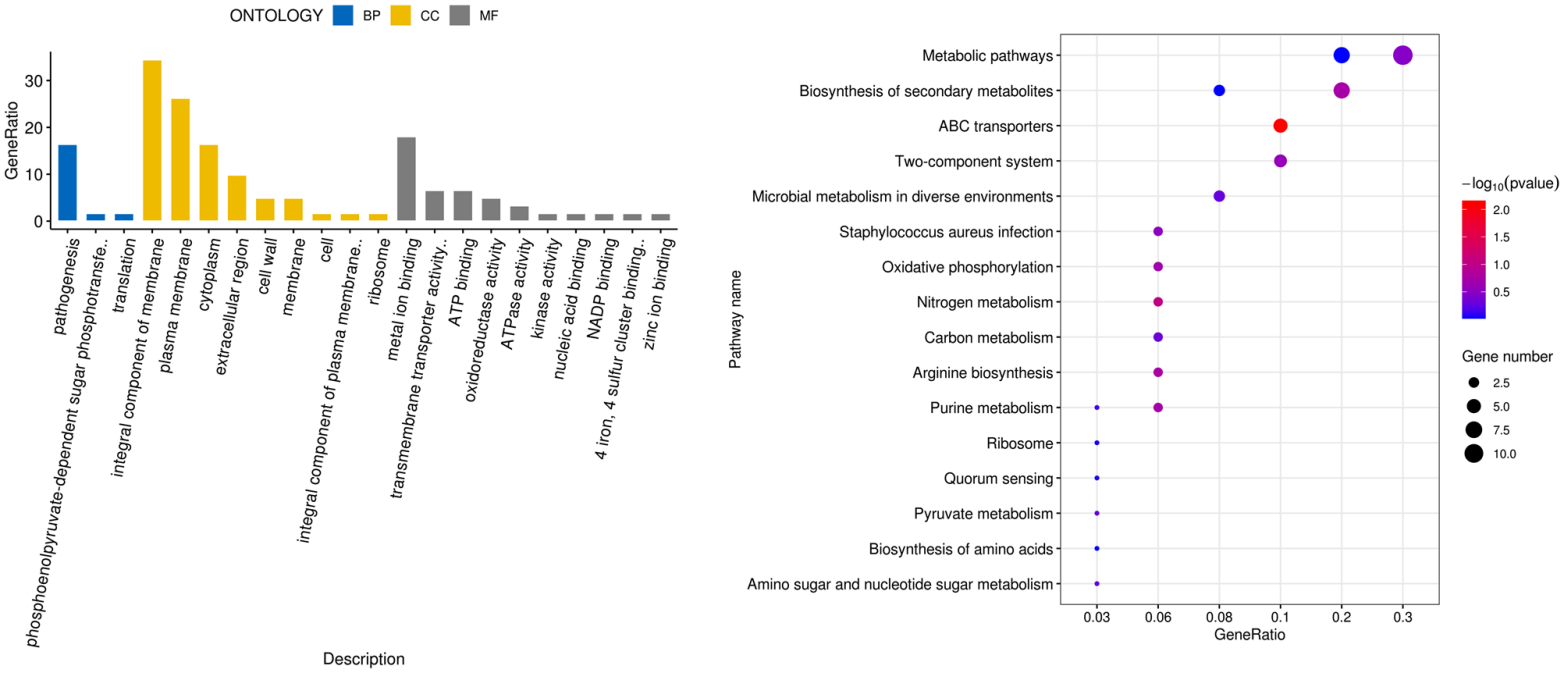
Functional and signaling pathway analysis of the overlapped DEGs according to BP, CC, MF, and KEGG pathways.

Enriched biological process (BP), cellular component (CC), and molecular function (MF) analyses were used to better understand the biological functions of overlapping DEGs. The results indicated that the significantly enriched GO term for BP was pathogenesis, the phosphoenolpyruvate (PEP)-dependent sugar phosphotransferase system (PTS) and translation. The significantly enriched GO terms for CC were the integral components of the membrane, plasma membrane, and cytoplasm. In addition, the significantly enriched GO terms for MF were metal ion binding, transmembrane transporter activity, and oxidoreductase activity.

In addition, the results of the KEGG pathway analysis revealed that these DEGs were primarily enriched in metabolic pathways, biosynthesis of secondary metabolite ABC transporters, and two-component systems.

Based on the results from the KEGG analysis, the effect of CT on *S. aureus* was concentrated on the metabolic pathways, moreover, the results of the GO-BP analysis indicated that the effect of CT on *S. aureus* concentrated on pathogenesis and the PEP-dependent sugar (PTS). PTS is a distinct system that is used by bacteria for sugar uptake when the energy source is phosphoenolpyruvate (PEP). In addition, PTS acts as a complex protein kinase system that regulates a wide variety of transport, metabolic, and mutagenic processes and the expression of numerous genes^6^. Among the DEGs, the pyk gene, which encodes PK was significantly downregulated. PK catalyzes the irreversible conversion of adenosine diphosphate (ADP) and PEP into adenosine triphosphate (ATP) and pyruvic acid, which are crucial for cellular metabolism. Therefore, PK plays a key role in controlling metabolic flux and ATP production. Meanwhile, as a highly conserved enzyme from animals to humans, PK has recently been identified as an essential gene for the survival of bacteria such as *Haemophilus influenzae, Streptococcus pneumoniae*, and *Mycobacterium tuberculosis*^7–10^. In addition, it has been reported that MRSA is inhibited in vitro by hampering the activity of PK^11–13^. Following CT action on *S. aureus*, PTS could detect changes in the surrounding environment that might further affect *S. aureus* metabolism via PK.

### MICs of vancomycin or CT against the MRSA strains

**Figure 3:**
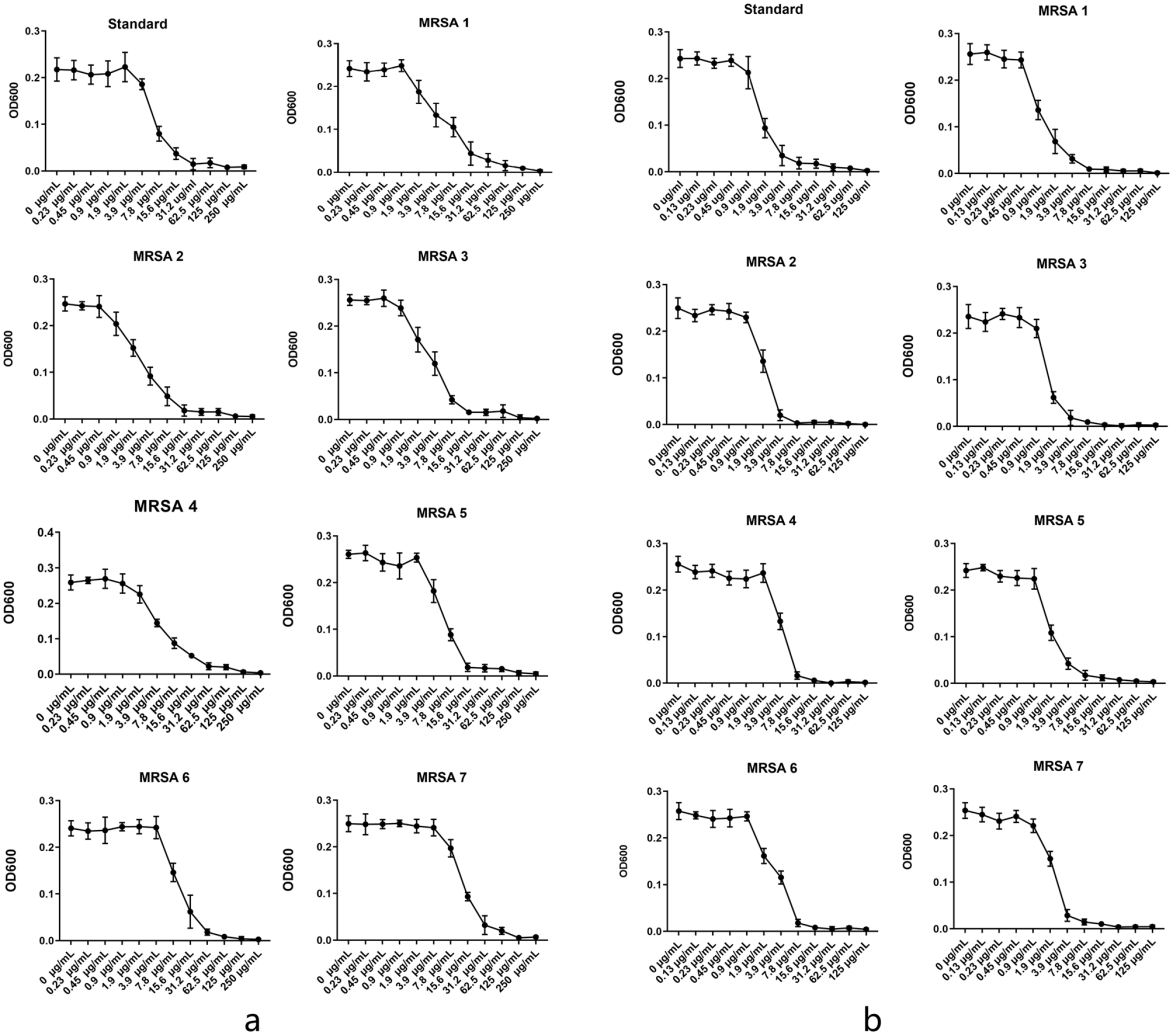
MIC assay of MRSA strains: (**a**) MICs of CT to seven MRSA strains isolated from clinical specimens and standard MRSA strain ATCC 43300; and (**b**) MICs of vancomycin to seven MRSA strains isolated from clinical specimens and standard MRSA strain ATCC 43300.

The MICs of vancomycin or CT against eight MRSA strains are given in Table 1 and Fig. 3. Among the seven clinical strains, five were derived from sputum and two were derived from blood, all of which were positive after a cefoxitin screening test. The MIC of vancomycin against ATCC43300 strain was 0.9 μg/mL, the MICs of vancomycin against seven other clinically isolated strains were between 0.45 and 1.9 μg/mL. This reflects the status of vancomycin as the first-line treatment for MRSA since the 1950s. The MIC of CT against a standard strain ATCC43300 was 1.9 μg/mL, the MICs of CT against seven other clinically isolated strains were between 0.9 and 3.9 μg/mL. This result showed that CT displays bacteriostatic action against MRSA, which agreed with previous reports^14^, which suggested that CT could be used to fight MRSA infection.

**Table 1:** Specimen and tests information on MRSA strains.

Strains	Source	Date of isolation (month and year)	Antibiotic resistances	Antibiotic sensitivity	V MIC (μg/mL)	CT MIC (μg/mL)
MRSA01	Sputum	04/2017	P, L, E, O, CIP, M, T, CL	G, TI, LINE, RI, V, TMP/SXT	0.45	0.9
MRSA02	Blood	04/2017	P, L, E, O, G, CIP, M, T, CL	TMP/SXT, TI, RI, LINE, V	0.9	0.9
MRSA03	Sputum	05/2017	P, G, L, E, O, CIP, M, T, CL	V, TMP/SXT, TI, RI, LINE	0.9	0.9
MRSA04	Sputum	07/2017	P, L, E, O, CIP, M, T, CL	TMP/SXT, TI, RI, V, LINE	1.9	1.9
MRSA05	Blood	07/2017	P, L, E, O, CIP, M, T, CL	TMP/SXT, TI, RI, LINE, V	1.9	1.9
MRSA06	Sputum	08/2017	P, L, E, G, O, CIP, M, T, CL	TMP/SXT, TI, RI, LINE, V	1.9	1.9
MRSA07	Sputum	08/2017	P, G, L, E, O, CIP, M, T, CL	TMP/SXT, TI, RI, LINE, V	0.9	3.9
ATCC43300	BJZY	–	–	–	0.9	1.9

*P* penicillin, *L* levofloxacin, *E* erythromycin, *O* oxacillin, *CIP* ciprofloxacin, *M* moxifloxacin, *T* tetracycline, *CI* clindamycin, *G* gentamicin, *TI* tetracycline, *LINE* linezolid, *RI* rifampin, *V* vancomycin, *TMP/SXT* trimethoprim–sulfamethoxazole, *BJZY* Beijing Zhongyuan Company, *V MIC* MIC of vancomycin to MRSA, *CT MIC* MIC of CT to MRSA.

### The effect of CT on the activity of MRSA PK

**Figure 4:**
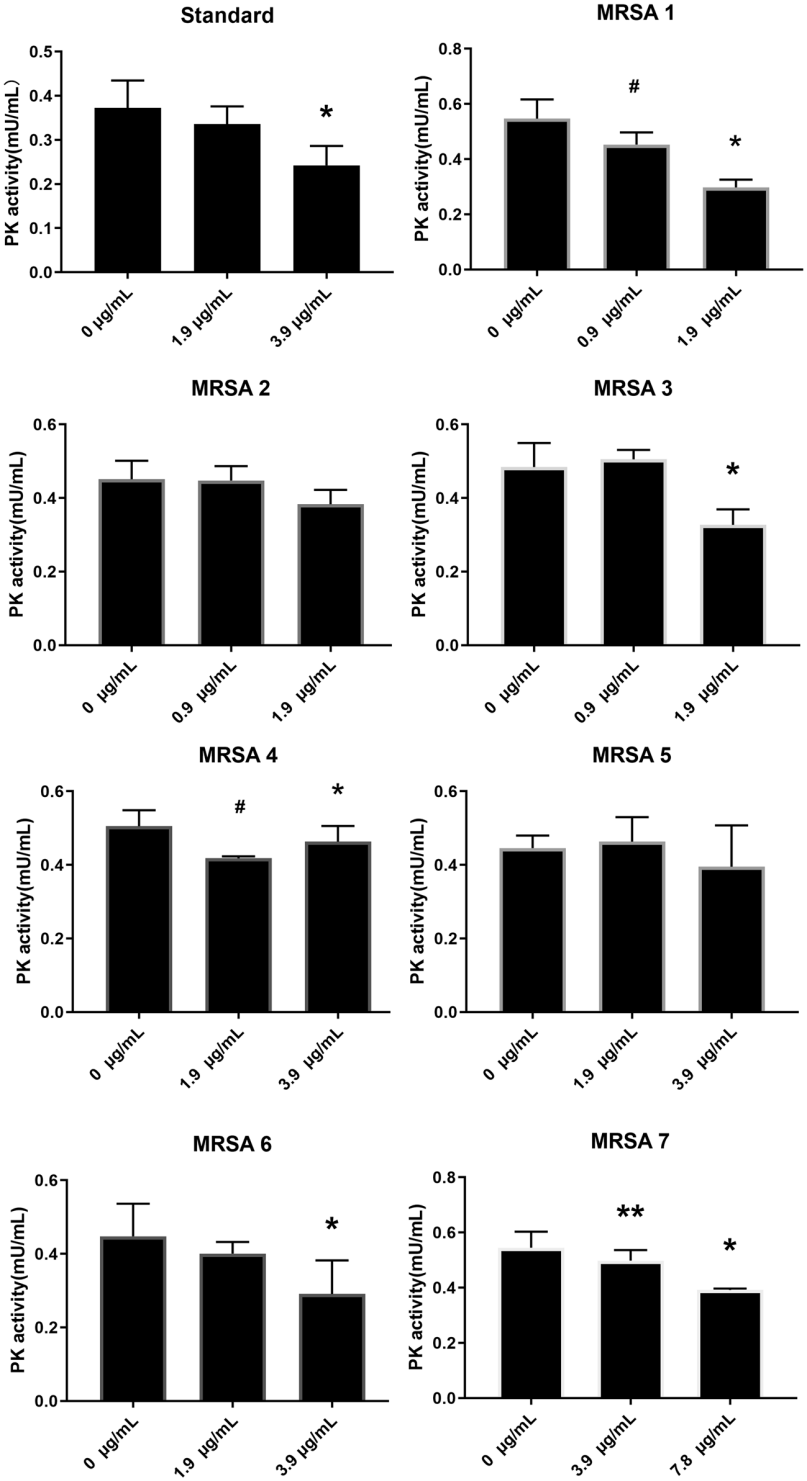
Effect of CT on the activity of PK of eight MRSA strains. ^#^Control group versus 1 × MIC group *p* < 0.05, *control group versus 2 × MIC group *p* < 0.05, **:1 × MIC group versus 2 × MIC groups *p* < 0.05.

In this study, the impact of CT was examined on the activities of PK in a standard MRSA strain ATCC43300 and seven other clinical strains. As shown in Fig. 4, in ATCC43300, MRSA3, and MRSA6, CT at a concentration of double the MIC reduced the activity of PK (*p* < 0.05). However, CT at the concentration of the MIC did not show an effect on the activity of PK. In MRSA1 and MRSA4, CT at the concentration of MIC and double the MIC reduced the activity of PK significantly (*p* < 0.05), with no difference between both concentrations. In MRSA7, CT at the same concentration and double the MIC decreased the PK activity with a difference between both concentrations (*p* < 0.05). In MRSA2 and MRSA5, CT did not affect PK activity at either concentration.

## Discussion

The evolution of MRSA demonstrates its genetic adaptation into a first-class multidrug-resistant pathogen. Following the introduction of penicillin and methicillin, *S. aureus* rapidly developed resistance to these β-lactam compounds^15^. In the 1950s, vancomycin was used to treat MRSA infections, but recently the emergence of MRSA strains that are less sensitive to vancomycin (VISA) or even resistant to vancomycin (VRSA) means that clinicians are less confident when dealing with MRSA. Therefore, there is an urgent requirement to explore effective drugs for MRSA^16^. Plants that grow in the natural environment are affected by pathogenic microorganisms, such as bacteria and viruses. Some plants have developed two major strategies to defend against pathogenic microorganisms: (1) plants have a lot of epidermal hairs, a thick waxy stratum corneum, and small stomata to block the invasion of pathogenic microorganisms; and (2) plants produce antibiotic substances, such as tannins and alkaloids to inhibit or kill invading pathogenic microorganisms^17^. Therefore, potential antimicrobial chemicals could be found in plants to treat pathogenic microorganisms, such as bacteria.

*Salvia miltiorrhiza* (Danshen) has been widely used in traditional Chinese medicine to treat a variety of diseases, which include coronary artery disease, acute ischemic stroke, hyperlipidemia, chronic renal failure, chronic hepatitis, and Alzheimer’s disease. In addition, Danshen has no serious adverse effects. The main bioactive constituents of *S. miltiorrhiza* include water-soluble phenolic acids and lipophilic diterpenoid tanshinones^18^. CT is one of the major tanshinones that were isolated from the roots of the Danshen plant. Recent studies have shown that CT has the potential to treat and prevent the previously mentioned diseases and it is a potent antibiotic agent^19^.

In this study, the antibacterial effect of CT on MRSA was tested in vitro, and the MICs of CT on MRSA reference strain ATCC43300 and seven clinical strains were detected using a two-fold serial dilution method. The results showed that CT displayed bacteriostatic action against MRSA, which was consistent with previous reports^14^, which suggests that CT could be used to fight MRSA infection.

Although thousands of herbal compounds have been listed as antimicrobial phytochemicals^20^, limited understanding of the mechanisms limits the application of these substances. To determine the mechanism of CT inhibition on the growth of MRSA, bioinformatics tools were used. In this study, the data from was retrieved from GSE13203 and systematically analyzed the differential gene expression of *S. aureus* were systematically analyzed between the CT treatment and control. Therefore, 64 DEGs were identified, which included 33 downregulated and 31 upregulated genes.

Then, GO and KEGG analysis of these DEGs revealed that CT had a greater impact on the metabolism of *S. aureus*. Because MRSA is a special species of *S. aureus* that carries a multidrug resistance gene, the metabolic pathways of MRSA and *S. aureus* are similar. As a facultative anaerobe, *S. aureus* uptakes a variety of nutrients that include glucose, mannose, mannitol, glucosamine, *N*-acetylglucosamine, sucrose, lactose, galactose, and beta-glucosides. The central pathways for glucose metabolism are the Embden–Meyerhof–Parnas pathway and the pentose phosphate cycle. Lactate is the end product of anaerobic glucose metabolism and acetate, and CO_2_ are the products of aerobic growth conditions^21^. In the glucose metabolic pathway, PK is the rate-limiting enzyme of energy metabolism, which plays a central role in the carbohydrate metabolism of MRSA. It catalyzes the final rate-limiting step of glycolysis. During this irreversible process, the high-energy phosphate bond in the PEP molecule is transferred to ADP to generate ATP. The metabolites PEP and pyruvate are related to other biosynthetic pathways, and therefore, the potential inhibition of PK could obstruct MRSA energy metabolism. The potential inhibition of PK might result in the decreased metabolism of MRSA^13,22–24^.

A PK test kit was used to detect the effects of CT on the activity of PK of MRSA in vitro*.* The results showed that except for in the MRSA2 and MRSA5 groups, CT hampered the PK activity of MRSA. PK was identified as a highly interconnected essential hub protein in MRSA, with structural features distinct from human homologs. Currently, the majority of antibiotics in use are directed at critical proteins that are unique to the bacteria and without human homologs to avoid mechanism based toxicity. In addition, based on the supposition that hub proteins are critical for bacterial survival and they should be very sensitive to mutations^25^, and therefore, targeting them should reduce the potential to develop resistant strains and species. Therefore, CT could be a valuable antibacterial candidate because of its effect on the PK of MRSA.

In this study, CT did not affect PK activity in MRSA2 and MRSA5, which were collected from a blood specimen and the five other strains were isolated from a sputum specimen. In different environments, *S. aureus* will utilize a variety of metabolic pathways, which depend on the oxygen content, source of carbohydrates, and other factors^26^. However, the MIC of CT was 0.9 μg/mL for MRSA2, and 1.9 μg/mL for MRSA5, which indicated that CT might have additional mechanisms that affect the growth of MRSA in addition to affecting the PK activity of both strains. As shown in Table 2, in addition to the pyk gene, a significant downregulation of isdB and isdG were observed, both of which belong to the iron-regulated surface determinant family. The GO-MF analyses revealed that CT affected the metal ion binding of *S. aureus*. Iron is vital for the growth and proliferation of nearly all organisms, including MRSA. In addition, iron is required for the colonization of host tissues by MRSA and subsequent pathogenesis^27^. Therefore, in addition to affecting PK activity, CT might inhibit MRSA growth by interfering with its iron metabolism, however, this requires further investigation.

**Table 2:** Top10 up and downregulated genes in DEGs.

Category	Gene symbol	Description	log_2_FC	Adjusted *p-*value
Upregulated	psuG	Pseudouridine-5′-phosphate glycosidase	2.332	0.077
	cydB	Cytochrome bd-I ubiquinol oxidase subunit II	2.029	0.011
	hrtB	Hemin transport system permease protein	1.784	0.022
	cap5C	Capsular polysaccharide biosynthesis protein Cap5C	1.780	0.063
	pflA	Pyruvate formate-lyase activating enzyme	1.737	0.142
	cydA	Cytochrome bd-I ubiquinol oxidase subunit I	1.653	0.018
	crtQ	Zeta-carotene desaturase	1.647	0.018
	clfA	MSCRAMM family adhesin clumping factor	1.640	0.018
	opuD2	Glycine betaine transporter 2	1.557	0.039
	crtM	Dehydrosqualene synthase	1.534	0.022
	psuG	Pseudouridine-5′-phosphate glycosidase	2.332	0.077
Downregulated	esaB	Type VII secretion protein	–2.607	0.011
	pyk	Pyruvate kinase	–2.285	0.164
	hutH	Histidine ammonia-lyase	–2.268	0.022
	isdB	Iron-regulated heme–iron binding protein	–2.106	0.025
	isdG	Staphylobilin-forming heme oxygenase	–2.006	0.020
	ribD	Bifunctional diaminohydroxyphosphoribosylaminopyrimidine deaminase/5-amino-6-(5-phosphoribosylamino) uracil reductase	–1.981	0.574
	gltS	Sodium/glutamate symporter	− 1.975	0.015
	nuc	Thermonuclease	− 1.960	0.022
	modA	Molybdate ABC transporter substrate-binding protein	− 1.860	0.057

*FC* fold change.

The most significant limitation of this study was that only changes in the activities of PK MRSA were examined after treatment with CT. Future research should include measuring the expression of PK of MRSA after treatment with CT. In addition, only seven MRSA strains were isolated from a clinical environment; therefore, a larger number of samples is required in future research to verify the antimicrobial mechanism of CT on MRSA.

In summary, CT inhibited the growth of MRSA in vitro. To determine the mechanism of activity, multiple bioinformatics tools were used combined with a comprehensive analysis of gene expression profiles to identify the key signaling pathways. CT interfered with the activities of PK, which is the key rate-limiting enzyme during glycolysis in MRSA. The results of this study might provide new insights into the antibacterial mechanisms of CT. However, further research and studies with larger sample sizes are required to confirm these findings.

## Methods

### Acquisition of gene expression profiling data

The microarray data for GSE13203 that was deposited by Feng et al.^28^ was retrieved from the GEO database(http://www.ncbi.nlm.nih.gov/geo/). The gene expression profile was generated using the Microarray Analysis Suite 5.0 (Affymetrix, Santa Clara, CA, USA). GSM333383, GSM333384, and GSM333385 were used for *S. aureus* specimens that were treated with CT for 45 m (intervention group). GSM333386, GSM333387, and GSM333388 were used are for the *S. aureus* specimens that were treated with saline for 45 m (control group).

### Raw data preprocessing and screening of DEGs

First, the chip data files were downloaded and the gene name information, sample number, and value were copied into a Microsoft Office Excel 2019 table (the Excel table cell format selected the text format) to sort the data and delete incomplete data, followed by saving all data as "input.txt". Then, gene expression differences between the CT treated samples and the control group were identified using the limma R package with the Empirical Bayes method^29^. The Benjamini–Hochberg false discovery rate (FDR) was used to correct for *p-*value. In this study, adjusted *p-*value < 0.05 and absolute Log_2_fold change (FC) > 2.0 were set as the cutoff criteria to screen DEGs. Furthermore, a volcano map was plotted with the ggplot2 package according to the adjusted *p-*value and log_2_FC. In addition, hierarchical clustering analysis of DEGs was performed and visualized using the pheatmap package in R Language.

### Functional and pathway enrichment analysis

To explore the biological functions and the pathways involved in the significant up and downregulated DEGs, Gene Ontology (GO) and Kyoto Encyclopedia of Genes and Genomes (KEGG) enrichment analyses were performed using the clusterProfiler package in R language^30^. GO is a comprehensive database that describes gene functions in three parts: BP, CC, and MF. GO function enrichment uses *p-*values < 0.05 as the threshold for significant enrichment. KEGG is a comprehensive database that integrates genomic, chemical, and system function information^31^. KEGG pathway enrichment uses *p-*values < 0.05 as the cutoff criteria for significant enrichment.

### Bacteria strains and materials

The MRSA reference strain ATCC43300 was purchased from Zhongyuan Company (Beijing, China). The seven clinical MRSA strains used in this study were isolated from the Microbiology Laboratory, Jiangsu Provincial Hospital of Traditional Chinese Medicine (Nanjing, Jiangsu Province, China) (Table 1). Mueller–Hinton Broth II (MHB II) and Mueller–Hinton agar were purchased from BD Biosciences (Sparks Glencoe, MD, USA). Stock solutions of varying concentrations were dissolved in dimethylsulfoxide (DMSO), which were purchased from Sigma-Aldrich (Shanghai, China).

### MICs

The minimum inhibitory concentrations (MICs) of CT or vancomycin against MRSA strain ATCC43300 and seven other clinical strains were determined in triplicate by broth microdilution or broth macrodilution using two-fold serial dilutions in MHB II, according to CLSI/NCCLS M100-S28^32^. The MICs were defined as the lowest concentration at which no visible growth was observed.

### The effect of CT on the activity of MRSA PK

PK activity was determined using a continuous assay coupled with lactate dehydrogenase (LDH), and the change in absorbance at 340 nm that was caused by NADH oxidation was measured using a SmartSpec Plus spectrophotometer (Bio-Rad, Laboratories (Shanghai), Hercules, CA, USA). The CT was diluted according to the MIC concentration of each MRSA strain and double the MIC concentration, with a control group of no CT treatment samples. In a 96-well plate, 50 μL of diluted CT was added in triplicate for each concentration. MRSA cells were harvested in the logarithmic growth phase and were diluted to a 0.5 turbidity standard bacterial suspension (1.5 × 10^8^ cfu/mL). A 96-well plate that contained 50 μL of bacteria suspension diluted in MHB II 1:500 in each well were mixed gently and placed in a 37 °C constant temperature incubator for 12 h. After the 96-well plate was removed from the incubator, 1 × 10^5^ cfu bacteria respectively were collected and centrifuged at 3000 rpm, at 4 °C for 5 m. After removing the supernatant, 100 μL of extract fluid (BC0540, Solarbio Science & Technology, Beijing, China) was added to the pellets and the bacterial cells were broken in an ultrasonic cell disruptor (Xinzhi JY92-IIN, Saide Electic, Hangzhou, Zhejiang Province, China). After centrifuging at 8000*g*, at 4 °C for 10 m, 30 μL of the supernatant was incubated with reagents (BC0540, Solarbio Science & Technology, Beijing, China) to initiate the reactions. PK activity proportional to the rate of change at 340 nm was expressed as specific activity (μmol/min/mg), which is defined as the amount of PK that catalyzes the formation of 1 μmol of either product per minute.

## Data Availability

The datasets generated during and/or analyzed during the current study are available from the corresponding author on reasonable request.
